# In‐line Raman spectroscopy real‐time glucose prediction method for commercial pneumococcal vaccine drug substance fermentation manufacturing process control

**DOI:** 10.1002/btpr.88532

**Published:** 2026-06-21

**Authors:** Griffin P. Thomas, Brian C. Marks, Barbara B. Alvarado‐Hernandez, Matthew Woodling, James O'Connor, Roberto Ortiz, John Higgins, Jon Shanter

**Affiliations:** ^1^ Vaccines and Advanced Biotechnologies Process Research and Development MRL, Merck & Co., Inc. Rahway New Jersey USA; ^2^ Analytical Enabling Capabilities MRL, Merck & Co., Inc. Rahway New Jersey USA; ^3^ Vaccine Drug Substance Commercialization, MMD Merck & Co., Inc. Rahway New Jersey USA

## Abstract

Traditional off‐line analysis methods to quantify residual metabolite concentrations in a drug substance fermentation process consume valuable time, require costly resources, and demand potentially hazardous manual operations when working with pathogenic biological organisms. This case study focuses on the successful method development and validation of Raman spectroscopy as an in‐line process analytical technology (PAT) to replace the existing off‐line assay for real‐time glucose quantification. The value and feasibility of Raman spectroscopy as an *in situ* PAT method are recognized and demonstrated as an enabling technology suited to mitigate challenges fundamental to lab‐scale process development and commercial manufacturing. Herein, application of chemometric techniques and multivariate analysis of Raman spectral data is described to develop a partial least squares model capable of predicting real‐time and accurate glucose concentrations with a root mean square error of prediction of 1.2 g/L. Through this work, the predictive Raman PAT glucose quantification method was validated to make process decisions in a commercially licensed Pneumococcal vaccine drug substance manufacturing facility for the first time at our company. The method was integral to the process control strategy and applied to accurately and robustly indicate the glucose concentration target ± 2 g/L for triggering subsequent process operations. Several advantages were realized through this work including advanced process control, reduced operating costs, and improved safety posture.

## 1 INTRODUCTION

Microbial fermentation processes are widely used in the biopharmaceutical industry as cultivation methods for the development and manufacture of numerous vaccine candidates.^1^ The rapid nature of some microbial fermentation processes renders both quantitative and qualitative analytics challenging.^2^ Glucose, a carbon source necessary to drive fermentation metabolism, is a common analyte of interest for quantification throughout a fermentation process.^3^

*Streptococcus pneumoniae* is a BSL2 (Biosafety Level 2) pathogenic microbial organism that constitutively expresses capsular polysaccharides that make up the dominant component of the cell's surface.^4^ The capsular polysaccharide plays a fundamental role in the virulence of the organism and is the primary antigen for pneumococcal vaccines. Our company's pneumococcal vaccine drug substance manufacturing process utilizes a fermentation leveraging glucose as the primary carbon source to cultivate *S. pneumoniae* to generate cell mass and the associated capsular polysaccharide. Glucose consumption rate and concentration monitoring are particularly important for our fermentation process. The glucose concentration is monitored during an initial batch phase and a target concentration is utilized to trigger process operations subsequent to the batch phase of the fermentation. In this way, the control strategy is defined to produce the highest amount of viable biomass at the end of the overall process. Once glucose concentration is within a target range, subsequent process steps are taken to progress the fermentation to completion. To monitor the rapid pace of glucose consumption throughout the fermentation, labor‐intensive and potentially hazardous operations are required to determine glucose concentration to advance the process. Manual samples are taken from the fermenter, manipulated in a biosafety cabinet to filter out the organism, and the supernatant is measured on a biochemical analyzer. These manual samples are slow, require routine offline equipment calibrations, additional labor, safety precautions, and increase the cost of goods for consumables. For these reasons, the necessity for a robust, scalable, in‐line analytical method capable of delivering real‐time glucose concentration measurements faster than offline methods was evident.

Raman spectroscopy, a vibrational spectroscopic technique, provides spectral fingerprints based on a molecule's bonding structure. This spectral data is interpreted through chemometric pre‐processing to build predictive models useful for monitoring analytes such as glucose throughout a fermentation process.^5^ Raman spectroscopy provides value in its ability to concurrently analyze multiple analytes in a non‐destructive nature through in‐line process culture contact within a fermenter, which effectively eliminates the need for manual sampling and subsequent processing.^5^ Its use has been demonstrated previously for bioprocesses. Some industry examples include a Raman method for glucose concentration monitoring throughout a yeast fermentation and a method to simultaneously quantify several analytes in a mammalian cell culture process.^6^, ^7^ Raman spectroscopy was an attractive method sought to remedy the challenges present with the existing offline glucose assay for our fermentation process. Rapid spectral acquisition, balancing noise and resolution to match the pace of glucose consumption in our complex and undefined fermentation medium, and validation of the method for inclusion in the control strategy for the commercial process were challenges navigated through development of the method. Following successful validation, the global Raman method was implemented for in‐process control for the first time at our company to indicate the progression from the batch phase of the fermentation for individual serotypes in the multi‐valent pneumococcal vaccine drug substance commercial manufacturing process (Figure 1).

**FIGURE 1:**
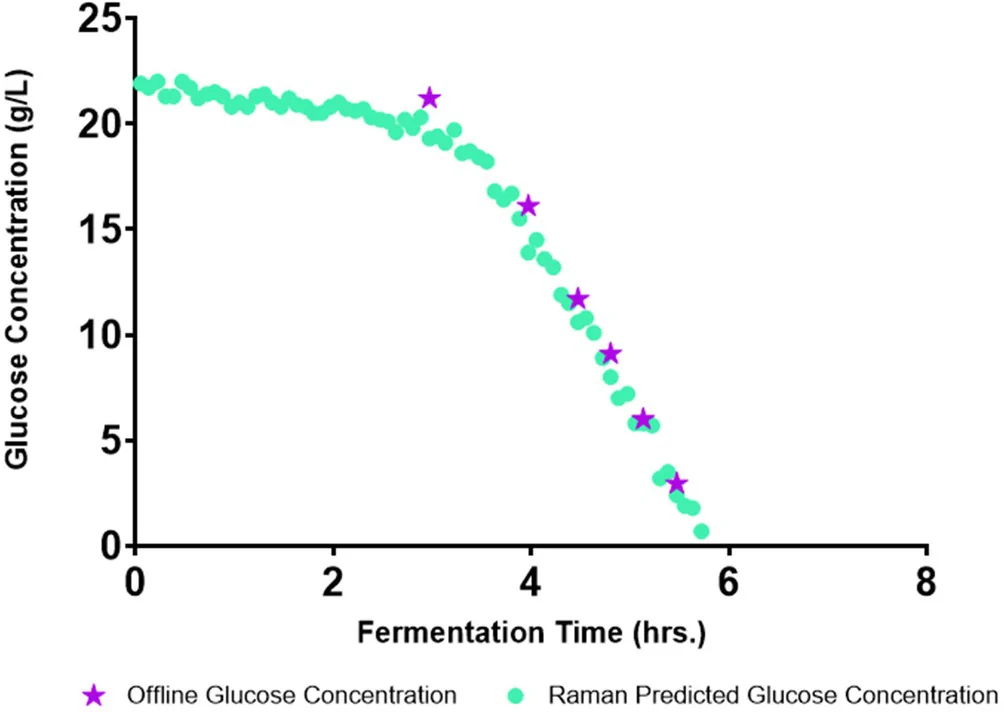
Raman prediction profile of glucose consumption for a commercial scale fermentation to inform batch progression and corresponding glucose offline manual samples.

## 2 MATERIALS AND METHODS

### 2.1 Microbial fermentation process description

The *S. pneumoniae* capsular polysaccharide manufacturing process consists of an initial cell expansion stage, followed by a production fermentation stage in a stirred‐tank bioreactor, and concludes with chemical inactivation and harvest prior to downstream clarification and purification. Both cell expansion and production fermentation stages use a chemically undefined complex medium comprised of glucose, salts, yeast extract, and soy peptone solutions.

The process begins with a cell expansion stage to increase biomass suitable for inoculation into the production fermenter. The production fermentation proceeds as a batch process and is maintained in a stirred‐tank bioreactor. Lab scale, pilot scale, and commercial scale bioreactors were used throughout development efforts. Temperature, agitation, air overlay, and pH are maintained through integrated automation as the fermentation progresses. As glucose is consumed by the organism throughout the fermentation, lactate is produced, and biomass accumulates through classical carbon utilization metabolic pathways.^8^ The fermentation process is typically monitored via manual samples from the bioreactor for subsequent offline assay on various analytical equipment. Each sample for glucose and lactate concentration measurement is filtered with redundant 0.22 μm sterilizing grade filters to remove the organism prior to measurement on the benchtop using a biochemical analyzer. Biomass is measured by optical density as absorbance units utilizing a UV–vis spectrophotometer at 600 nm on an unfiltered sample in a capped cuvette. Once glucose concentration is within range of the process target, the batch is progressed further until it ultimately undergoes chemical inactivation to ensure kill of the organism is achieved. After a period of mixing with temperature control, the batch is harvested for further downstream processing and purification. The fermentation process is portrayed in Figure 2.

**FIGURE 2:**
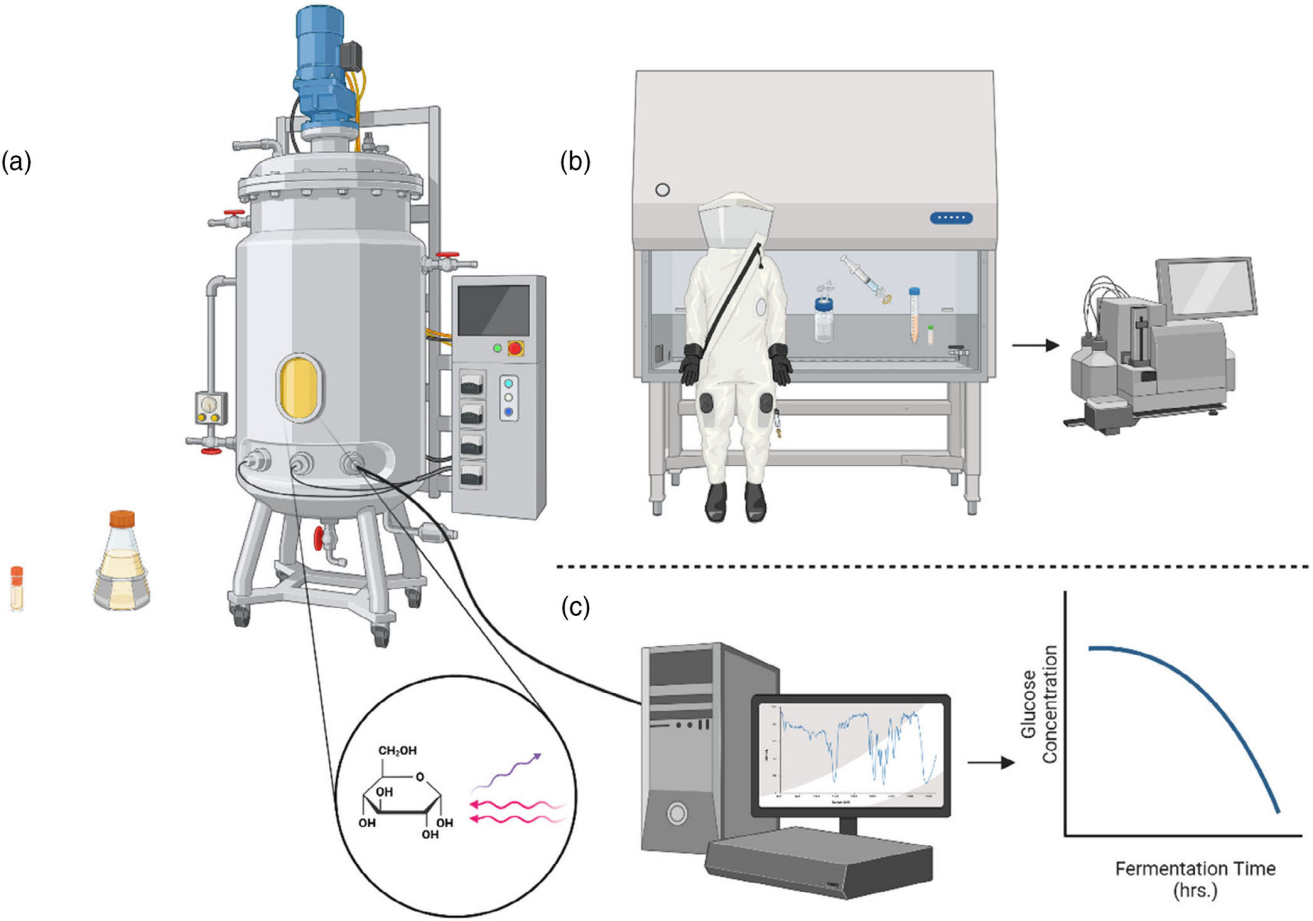
(a) The process consists of a cell expansion stage followed by a production fermentation in a stirred‐tank fermenter. (b) Manual samples are routinely removed from the fermenter, processed inside of a biosafety cabinet, and measured on a benchtop biochemical analyzer to provide glucose concentration. (c) In‐line Raman spectroscopy is implemented to replace existing offline glucose assay to provide real‐time glucose measurements.^9^

### 2.2 Spectral data acquisition

Raman spectral data was collected at three separate laboratories and manufacturing facilities of our company. Endress+Hauser Optical Analysis Raman Rxn2 analyzers were used for laboratory scale spectral data collection. Endress+Hauser Optical Analysis Raman Rxn4 analyzers were used at pilot and commercial scale data collection. Both analyzers provide a 400‐mW excitation laser with a wavelength of 785 nm suitable for fermentation processes. The detector of the Raman analyzer provides spectral coverage of 100–3425 cm^−1^. Acquisition settings included a 5000‐ms exposure with 36 accumulations resulting in a 3‐min scan per spectrum, which is programmed to repeat at 5‐min intervals. In laboratory settings, four bioreactors were monitored providing predictions every 20 min per bioreactor. At manufacturing scale, a single channel is used providing predictions every 5 min.

The Endress+Hauser Optical Analysis Raman Rxn2 systems were fit for four channel operation whereas the Endress+Hauser Optical Analysis Raman Rxn4 analyzer was fit for single channel operation. Fully Steam in place/Clean in Place (SIP/CIP) capable Endress+Hauser Optical Analysis Rxn‐45 probes were utilized for each channel. Each probe was placed in the side port of the production fermenter submerged in the culture and protected from ambient light for spectral data acquisition. The Endress+Hauser Optical Analysis Rxn2 and Rxn4 systems were controlled by the PAT data collection and management system, SIEMENS SIPAT. SIPAT was capable of both collecting and archiving spectral data. A SIMCA calculation engine was integrated within SIPAT to provide real‐time analyte predictions.

### 2.3 Model development and validation strategy

A failure mode and effects analysis (FMEA) risk assessment was performed to identify potential failure modes that could render the Raman instrument unfit to measure relevant process attributes, such as glucose, accurately and reliably. Additionally, this assessment was performed to determine which factors could impact the validation of the method. A degree of severity score, a probability of occurrence score, and an ability to detect score were applied to each potential failure mode event. Response studies were performed to build a data package for inclusion in the calibration set during development to address and reduce the probability of the identified failure modes.

Following FMEA scoring, the primary focus during the development of the model was to collect spectral data on fermentation batches where glucose concentrations were outside the typical process ranges. This was achieved through the addition of non‐routine glucose boluses at select times throughout the fermentation to intentionally challenge predictive model correlations as glucose was consumed by the organism. Furthermore, spectral data was collected on batches using fermentation medium incorporating various lots of raw materials to identify sensitivities or biases from the raw material input. Additionally, fermentation batches were intentionally controlled outside of typical process ranges for pH (± 0.2), temperature (± 2°C), and agitation (+50/−95 RPM) to identify bioreactor parameter impacts on the model. Raman spectral data was also collected using several spectrometer base units, fiber optic cables, and probes to aid in building and improving robustness into the method when using Raman at different labs, facilities, and scales.

The Raman glucose method was validated for specificity, accuracy, linearity, range, measurement precision, and robustness. The validation strategy followed a data stratification approach. This approach ensured that the model was developed with a calibration set using data comprised of as much instrument, process, and analytical variation as possible. A validation data set was then collected to appropriately challenge that variability within the calibration data set.

Approximately one hundred fermentation batches were available for model development and validation. Data was collected for several different serotypes of *S. pneumoniae* using eight different bioreactors and five different Raman instruments at both lab and commercial scale. Throughout the fermentation of these development batches, approximately 8–12 offline samples were taken from the bioreactor at the time of Raman spectral data acquisition to generate glucose reference values in grams of glucose per liter of fermentation broth.

Over five hundred offline reference samples were included in the development and validation data sets. The fermentation batches were divided into subsets of calibration and validation sets to validate the model on independent batches. At least three fermentation batches of each serotype were included in the validation set.

## 3 RESULTS AND DISCUSSION

### 3.1 Model development

Chemometric analysis was performed on the Raman spectral data using Sartorius SIMCA software to develop the predictive partial least squares glucose model. Multiple spectral pre‐processing steps, such as standard normal variate (SNV), first and second derivatives calculated with second order polynomial, and Savitzky–Golay (SG) algorithm using smoothing windows of 15 or 25, were used. Combinations of these pre‐processing steps across different spectral regions were evaluated during development with the goal of establishing robustness and model predictability. Spectral regions and pre‐processing treatments were evaluated using root mean square error of prediction (RMSEP). The model with the lowest predictive errors was selected.

The selected model applies spectral pre‐processing using SG second order derivative to remove offsets, linear baselines, and noise, then SNV to normalize each spectrum and reduce multiplicative scatter effects and baseline offsets, and finally mean‐centering to the combined spectral regions 415–1750 cm^−1^ (fingerprint region) and 2650–3150 cm^−1^ C–H (carbon–hydrogen) stretch. Regions excluded from pre‐processing contained interference from the immersion probe window at 406 cm^−1^, disjointed spectral features around 1850 cm^−1^, and regions of spectral variations that were not relevant to analyte concentration.

In the case of our *S. pneumonaie* fermentation process, the organism exhibits the homolactic fermentation pathway whereby glucose is consumed, metabolized to pyruvate, and subsequently converted to lactate via lactate dehydrogenase. The presence of lactate in the process culture presented anticorrelative spectral bands, such as 855 cm^−1^, and were considered for removal during variable selection to maintain specificity for glucose. It was not possible to eliminate all lactate spectral bands, particularly in the 2650–3150 cm^−1^ region which corresponds to the C–H stretch region, and was included to obtain desired model performance.

After considering variable importance in projection (VIP) scores and stepwise selection, spectral regions 1053–1186 cm^−1^, 1352–1437 cm^−1^, and 2860–3100 cm^−1^ were chosen, as seen in Figures 3 and 4. In Figure 4 spectral feature changes can be observed at different glucose concentrations in both media without culture and during the fermentation process. The resulting model consisted of a single latent variable. The model was then validated considering the following acceptance criteria: Specificity, Accuracy, Linearity, Range, Precision, and Robustness.

**FIGURE 3:**
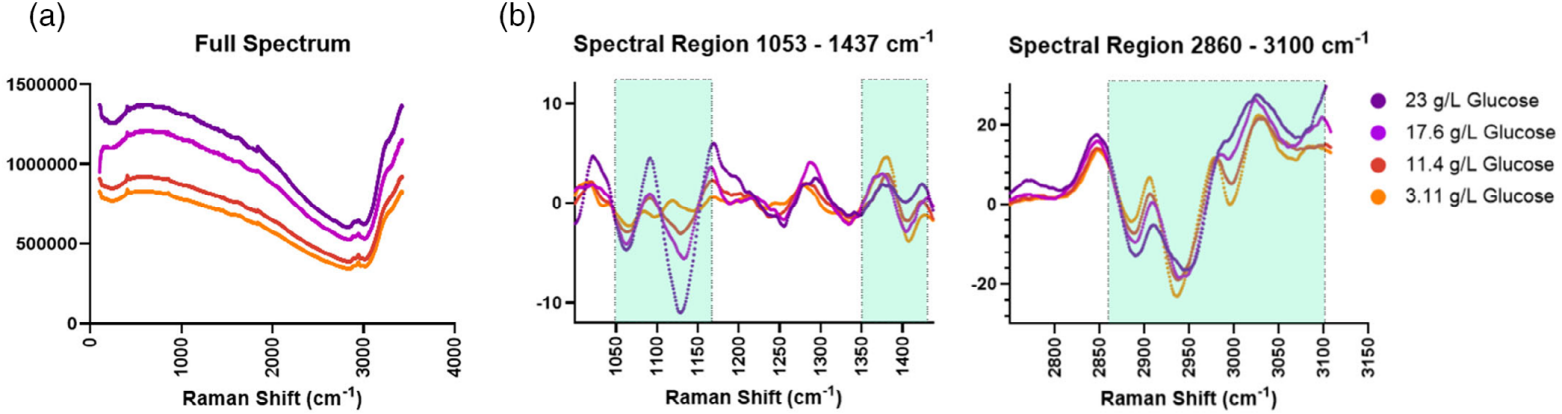
Raw (a) and pre‐processed (b) spectral data of key regions included in the model.

**FIGURE 4:**
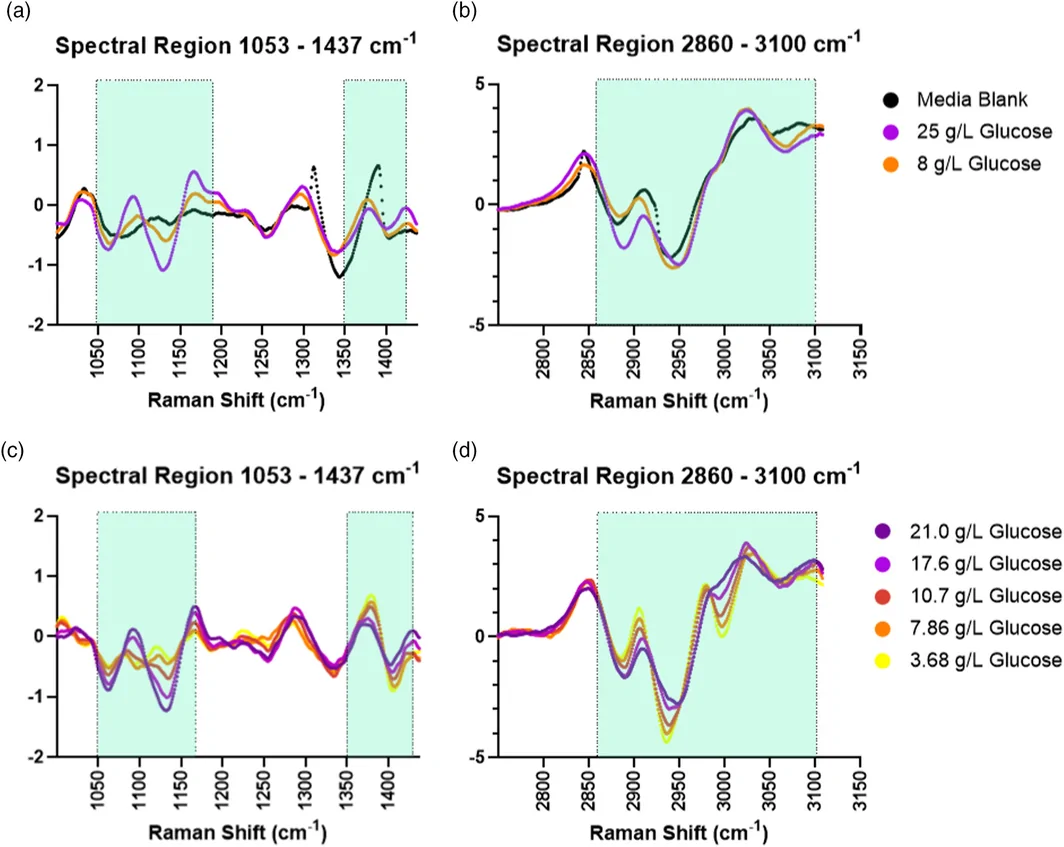
Pre‐processed Raman spectral data of key regions showing feature evoluation as glucose concentration changes in medium (a) and (b) and during the fermentation (c) and (d).

### 3.2 Model validation

The chemometric model was externally validated using an independent validation data set consisting of Raman spectral batch data not included in the calibration data set to predict glucose concentration. The Raman glucose predictions obtained were compared to offline measured glucose concentrations. RMSEP was the primary method to assess the reliability and predictability of the model. Sources of error contributing to RMSEP include the inherent accuracy and precision of the reference method and changes to analyte concentration during gaps in the timing of offline sample collection and inline spectrum collection. RMSEP is calculated using the following equation:

$$\text{RMSEP}=\sqrt{\frac{\sum_{i=1}^{n}{\left({y^{i}}_{\text{measured}}-{y^{i}}_{\text{predicted}}\right)}^{2}}{n}}$$

where *n* equals the number of samples, and *y* ^*i*^_measured_ represents the offline reference measured glucose concentration and *y* ^*i*^_predicted_ represents the Raman predicted glucose concentration.

Model outlier diagnostic tools were used to justify removal of atypical spectra from calibration and validation sets. The same outlier threshold values are applied in real‐time to prevent predictions on atypical spectra. A spectrum is considered an outlier if its distance to the model in the X space (DModX) is greater than 2.5 times the critical limit (DCrit) or is outside the model's 99% confidence region calculated using Hotelling's *T* ^2^. Real‐time predictions are also flagged if they are outside the validated concentration range.

The acceptance criteria of the Raman method were evaluated, considering the elements of specificity, accuracy, measurement precision, linearity, and robustness across multiple instruments and serotypes. Table 1. shows the acceptance criteria evaluation of the model. Specificity is maintained by selecting for wavelength regions where glucose has Raman signal discernable from negative controls. For accuracy, two ranges were considered; the entire glucose concentration range of 0–26 g/L is useful for trending while a narrower range of 0–10 g/L is relevant for in‐process control for decision making for progression of the fermentation. An RMSEP ≤1 g/L glucose within this in‐process control range offers sufficient accuracy that the difference in batch progression trigger time is negligible between the offline biochemical analyzer and Raman. Subsequent model verification studies demonstrated that the difference between using the offline biochemical analyzer and the Raman method for batch progression trigger time was less than 20 min. Linear regression acceptance criterion of the glucose model was required to have *R* ^2^ ≥0.9, the model proved to have a *R* ^2^ of 0.959 meeting this criterion. The full range of the assay was established by the sample with highest and lowest glucose values in the validation set that were within the calibration range and meet the acceptance criteria for linearity and/or accuracy, as seen in Table 1. As a dynamic system, method precision is determined with consecutive Raman measurements of the pre‐inoculation tank while the glucose concentration is constant, the highest RSD% observed was of 2.5% showing good precision. Finally, for method robustness, the RMSEP for individual serotypes and Raman instruments in the validation set is ≤3 times the validation acceptance criteria specified for method accuracy, Table 1 shows the model meets the Robustness acceptance criteria.

**TABLE 1:** Method validation results.

Validation element	Validation results
Specificity	The method uses glucose Raman signals discernable from water and blank fermentation media controls
Accuracy	1–26 g/L Glucose Range RMSEP = 1.2 g/L Glucose 1–10 g/L Glucose Range RMSEP = 1.0 g/L Glucose
Linearity	*R* ^2^ = 0.959
Method range	1–26 g/L Glucose
Measurement precision	Highest observed RSD = 2.5%
Robustness	Individual serotypes RMSEP = 1.0–1.7 g/L Glucose Individual Raman instruments RMSEP = 1.1–1.3 g/L Glucose Variation in fermentation pH, temperature, and agitation did not trend with prediction error

### 3.3 Model performance verification and lifecycle management

Model lifecycle management ensures the method continues to perform as intended for the established control strategy. Routine verification is performed for every prediction by using outlier diagnostics with pre‐established thresholds. For periodic verification, probe replacement was identified as a higher risk activity that warranted model verification by parallel testing with the biochemical analyzer. Model performance is verified by using acceptance criteria in conformance to accuracy used for validation, and elements of precision and linearity are calculated for trending. Model health can be continually assessed by trending outlier diagnostic values and periodic verification results, which can be facilitated by automated reports of the batch data.

Periodic verification was conducted across three commercial scale fermentation batches of different serotypes. Seven manual samples were taken from the batch fermentation phase of each lot and analyzed on the offline biochemical analyzer for comparison to the Raman glucose prediction method. Visual representations of the method's performance are available in Figure 5. Further analysis of the data including RMSEP is provided in Table 2. The offline glucose concentrations compared to the Raman predicted glucose concentrations of these batches resulted in an RMSEP of 0.94 g/L glucose. The RMSEP calculated in the verification exercise is less than the defined RMSEP of the validation acceptance criteria. As such, the method was deemed to be performing as intended and remained suitable for use.

**FIGURE 5:**
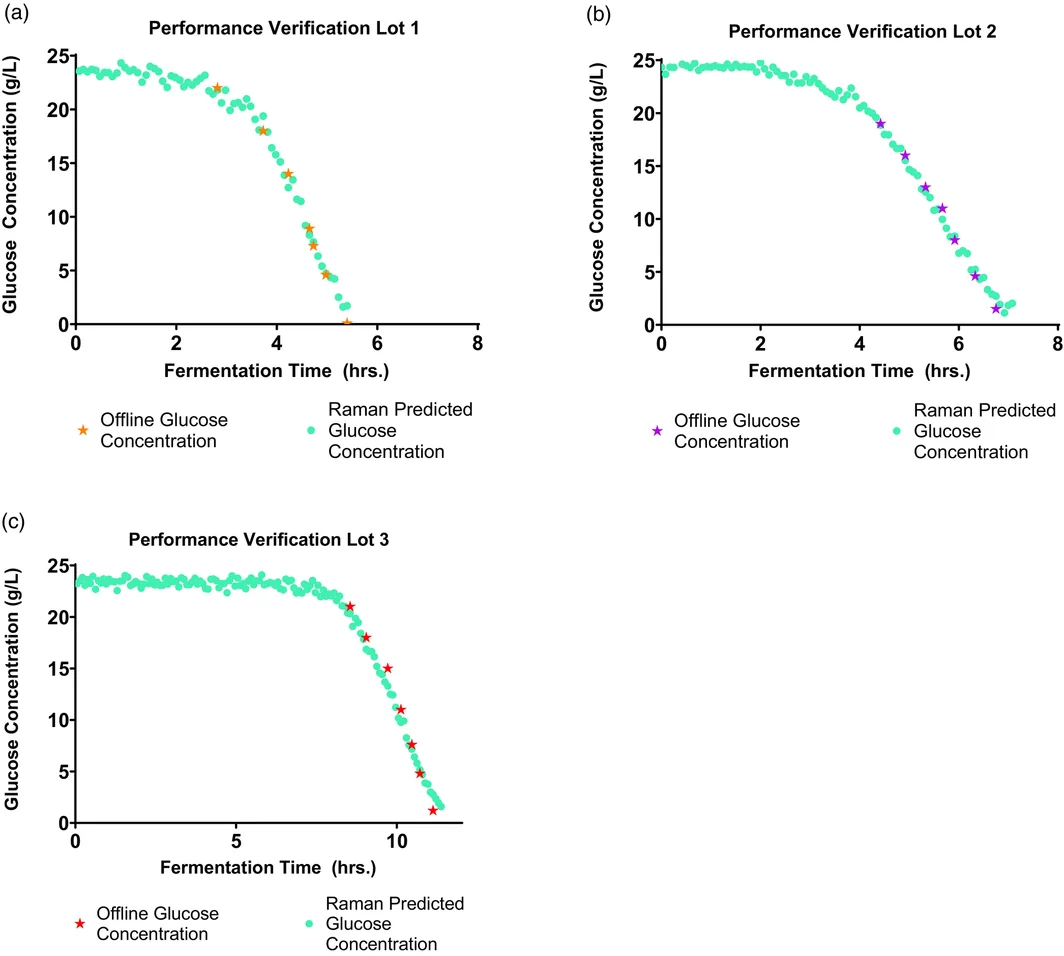
Raman glucose method predictions compared to offline samples for three lots used to verify model performance.

**TABLE 2:** Model performance verification.

Fermentation lot	Raman glucose concentration (g/L)	Offline glucose concentration (g/L)
Performance verification lot 1	22	22
	19	18
	13	14
	8.3	8.9
	7.7	7.3
	4.7	4.6
	1.7	0.086
Performance verification lot 2	19	19
	16	16
	13	13
	10	11
	8.4	8.0
	5.2	4.6
	2.7	1.5
Performance verification lot 3	20	21
	17	18
	13	15
	9.8	11
	7.2	7.6
	5.2	4.8
	2.8	1.2
	RMSEP	0.94

The ability of the method to repeatedly and accurately provide Raman glucose predictions at the end of the batch phase of fermentation within a specified glucose concentration range demonstrates the robustness of the process control strategy. In a sample size of 26 commercial scale batches, all were progressed within ± 1 g/L glucose of the target concentration when implementing the Raman PAT method. This is then compared to a sample size of 13 commercial scale batches using the traditional offline analysis method. In this data set, the batches were progressed across a comparatively wider range of ± 3 g/L glucose of the target concentration (Figure 6). This analysis further supports the accuracy and reliability of the Raman method across multiple batches and displays improved robustness to make process decisions following commercial scale implementation.

**FIGURE 6:**
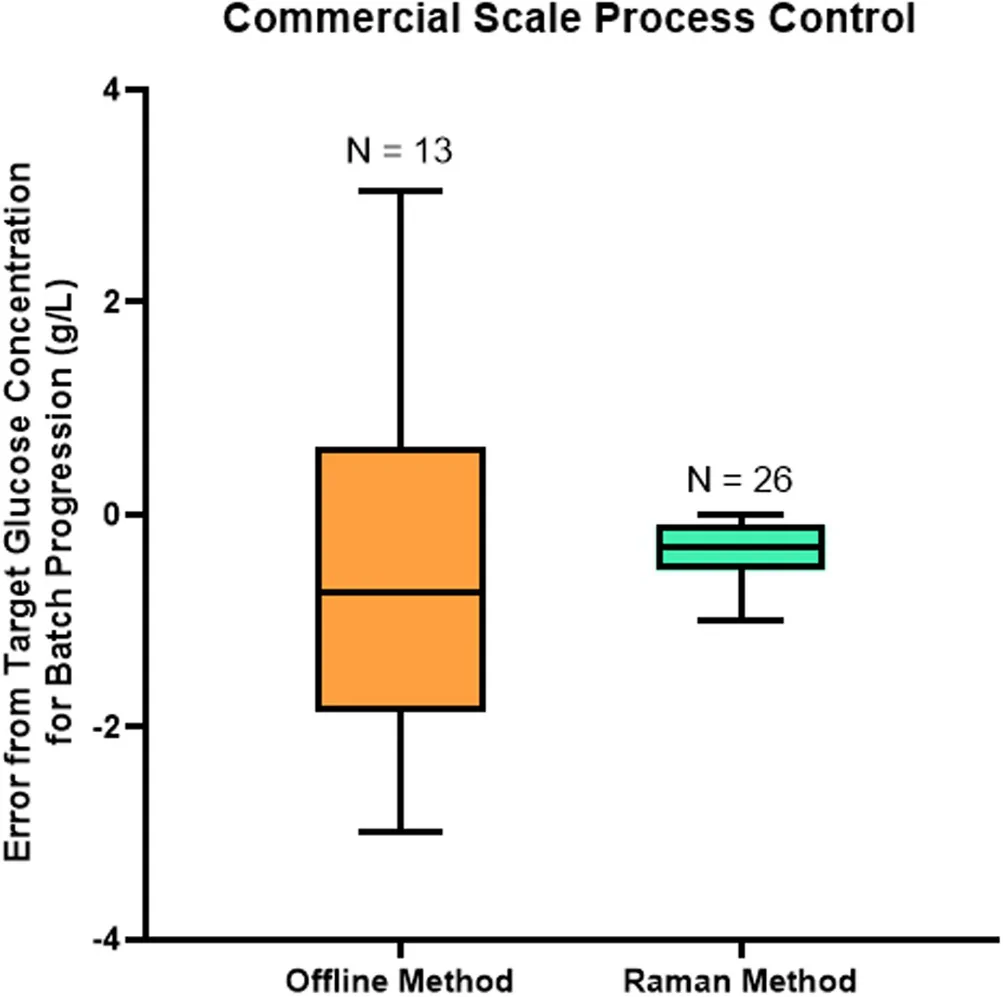
Error from Raman method target glucose concentration for batch progression compared to traditional offline method of commercial scale batches.

## 4 CONCLUSIONS

The validated Raman glucose method was considered suitable for its intended use as an alternative to the existing offline sample analysis method on the biochemical analyzer to measure glucose concentration throughout the batch fermentation process. Accordingly, the Raman glucose method was implemented during a performance verification series to indicate the progression trigger of each fermentation lot. Following the successful demonstration of the method, offline samples were no longer required for making process decisions. The Raman glucose method was formally incorporated into the control strategy for commercial manufacture of the Pneumococcal polysaccharide drug substance. This Raman glucose method was the first application of real‐time Raman PAT to make process decisions in Merck's large molecule commercial manufacturing and set the precedent for further large molecule vaccine pipeline programs. Several advantages were realized through implementation, including eliminating the need to handle offline samples of BSL2 material, improving process robustness of an in‐process control, reducing process downtime, and improving available process characterization data.

## AUTHOR CONTRIBUTIONS

**Barbara B. Alvarado‐Hernandez:** Writing – review and editing; data curation; methodology. **Griffin P. Thomas:** Conceptualization; investigation; writing – original draft; methodology; validation; visualization. **Roberto Ortiz:** Writing – review and editing; project administration; supervision. **Jon Shanter:** Writing – review and editing; project administration; supervision. **Matthew Woodling:** Writing – review and editing; project administration; supervision. **Brian C. Marks:** Conceptualization; investigation; writing – original draft; methodology; validation; visualization; software; data curation. **John Higgins:** Writing – review and editing; project administration; supervision. **James O'Connor:** Conceptualization; investigation; methodology; validation; writing – review and editing.

## FUNDING INFORMATION

Research was funded by Merck Sharp & Dohme LLC, a subsidiary of Merck & Co., Inc., Rahway NJ, USA. Authors may at times hold stock and/or options in Merck & Co., Inc., Rahway NJ, USA.

## CONFLICT OF INTEREST STATEMENT

Authors are employees of Merck Sharp & Dohme LLC, a subsidiary of Merck & Co., Inc., Rahway NJ, USA.

## Data Availability

The data that support the findings of this study are available on request from the corresponding author. The data are not publicly available due to privacy or ethical restrictions.
